# A Scoping Review of Cognitive Training in Neurodegenerative Diseases via Computerized and Virtual Reality Tools: What We Know So Far

**DOI:** 10.3390/brainsci11050528

**Published:** 2021-04-21

**Authors:** Stefano Lasaponara, Fabio Marson, Fabrizio Doricchi, Marco Cavallo

**Affiliations:** 1 Department of Psychology, Sapienza University of Rome, 00185 Rome, Italy; stefano.lasaponara@uniroma1.it (S.L.); fabrizio.doricchi@uniroma1.it (F.D.); 2 Department of Human Sciences, LUMSA University, 00193 Rome, Italy; 3 Research Institute for Neuroscience, Education and Didactics, Fondazione Patrizio Paoletti, 06081 Assisi, Italy; f.marson@fondazionepatriziopaoletti.org; 4 Department of Human Neuroscience, Sapienza University of Rome, 00185 Rome, Italy; 5 Department of Neuropsychology, IRCCS Fondazione Santa Lucia, 00179 Rome, Italy; 6 Faculty of Psychology, eCampus University, 22060 Novedrate, Italy; 7 Clinical Psychology Service, Saint George Foundation, 12030 Cavallermaggiore, Italy

**Keywords:** Alzheimer’s disease, cognitive impairment, frontotemporal dementia, multiple sclerosis, neuropsychology, Parkinson’s disease

## Abstract

Most prevalent neurodegenerative diseases such as Alzheimer’s disease, frontotemporal dementia, Parkinson’s disease and multiple sclerosis are heterogeneous in their clinical profiles and underlying pathophysiology, although they typically share the presence of cognitive impairment that worsens significantly during the course of the disease. Viable pharmacological options for cognitive symptoms in these clinical conditions are currently lacking. In recent years, several studies have started to apply Computerized Cognitive Training (CCT) and Virtual Reality (VR) tools to try and contrast patients’ cognitive decay over time. However, no in-depth literature review of the contribution of these promising therapeutic options across main neurodegenerative diseases has been conducted yet. The present paper reports the state-of-the-art of CCT and VR studies targeting cognitive impairment in most common neurodegenerative conditions. Our twofold aim is to point out the scientific evidence available so far and to support health professionals to consider these promising therapeutic tools when planning rehabilitative interventions, especially when the access to regular and frequent hospital consultations is not easy to be provided.

## 1. Introduction

Neurodegenerative diseases such as Alzheimer’s disease (AD), Frontotemporal Dementia (FTD), Parkinson’s disease (PD) and Multiple Sclerosis (MS) are a public health priority throughout the world with tremendous medical, psychological and economic repercussions. Their prevalence and incidence had dramatically increased with age over the last decades, and they are expected to continue to grow due to the gradual rise in the average length of life in most countries [1]. Neurodegenerative diseases are heterogeneous in their clinical profiles and underlying pathophysiology, although they typically share the presence of significant cognitive impairment. Time and accuracy of diagnosis are crucial factors, as they would allow the planning of timely and appropriate management of cognitive deficits. As no effective pharmacological treatments for cognitive symptoms in this clinical domain are currently available, in recent years, various studies had started to investigate the potential contribution of Computerized Cognitive Training (CCT) and Virtual Reality (VR) tools in contrasting patients’ cognitive decay (see for example the systematic review by Hill and colleagues [2], with a specific focus on mild cognitive impairment and dementia). Even if the use of these remote tools was considered as a promising therapeutic option from the very beginning, the severe COVID-19 pandemic has made it evident the essential need of planning remote interventions implemented by patients and caregivers without the need for them to go repeatedly to hospitals and clinics.

After presenting the most prominent neuropsychological features of the major neurodegenerative diseases here considered, the present in-depth review reports the state-of-the-art of CCT and VR studies targeting cognitive impairment in these clinical conditions. Our twofold aim is to consider what had been done so far in the field and to highlight how these therapeutic options can help health professionals to manage more effectively the cognitive deficits that characterize patients’ profile while reducing significantly the number of visits to clinics.

### 1.1. Neuropsychological Profiles of Main Neurodegenerative Diseases

Alzheimer’s disease (AD) is a highly disabling neurodegenerative disorder that represents more than 60% of dementia diagnoses among the elderly worldwide. Neurophysiology of AD is mainly characterized by the extra-cellular accumulation of amyloid-β peptide plaques and intracellular neurofibrillary tangles containing phosphorylated tau protein on cortical and sub-cortical regions [3]. These physiological abnormalities undermine cerebral integrity, causing global white and grey matter atrophy involving frontal regions, cingulate and temporal cortex and praecuneus, selective hippocampal atrophy and increased ventricular volume [4,5]. However, the connection between increasing amyloid plaques and the manifestations of main cognitive deficits that typically characterise patients’ profile seems to be not so direct, as treatments aimed to reduce amyloid accumulation are relatively useless in contrasting cognitive decline [6,7]. The first neurophysiological abnormalities affect medial temporal lobe structures involved in memory [8], semantic retrieval [9] and spatial processing [10]. As a result, early cognitive changes typically involve progressive memory loss, reduced visuo-spatial attention and topographical disorientation [11,12,13], especially in early-onset AD [14]. Then, the disease progression spreads on a large scale [15], causing anterograde amnesia [16] and significant impairment in the realm of executive functions [17]. During the early stages of the condition, it is not trivial to differentiate AD from pre-clinical manifestations that require attention such as Mild Cognitive Impairment (MCI) [18,19].

AD cognitive deficits and comorbidity with neuropsychiatric disorders such as depression, anxiety, aggressiveness and disinhibition heavily affect Quality of Life (QoL) of both patients and caregivers [20,21]. Preservation of autonomy in performing an instrumental activity of daily living and intervention to improve cognitive functions seem to directly affect improving QoL scores [20,22]. A definitive cure for AD has not been found yet, since aetiology is still unknown and pathogenesis unclear [23]. For this reason, main therapeutic protocols can only try to attenuate disease progression by reducing symptoms or delaying their onset to maintain a sufficient level of physical, psychological and social functioning [24]. Usually, such interventions are designed to improve individual goal-directed behaviour and require the active participation of caregivers and professionals [25].

Fronto-temporal dementia (FTD) is a neurodegenerative disorder which shares various clinical aspects with AD. Differential diagnosis is not trivial in the earliest stages of disease’s progression, and cognitive screening tools such as the Mini-Mental State Examination (MMSE) are not sensitive enough to differentiate between FTD and AD [26]. Main differences in the behavioural domain regard the loss of social and personal awareness and the presence of stereotyped repetitive behaviours in FTD [27,28], while on the cognitive side, it lacks significant memory deterioration. Episodic and autobiographical memories are relatively well preserved since no hippocampal deterioration has been observed in FTD, especially in the early stages [29,30]. Importantly, FTD develops earlier and progresses faster than AD [31,32], making an efficient diagnostic process even more crucial for this condition. Clinical manifestations of FTD are heterogeneous and include a first distinction between a behavioural variant (bvFTD) and primary progressive aphasia (PPA), with the latter further divided in semantic (svPPA), non-fluent (nfvPPA) and logopenic (lvPPA) variants. The behavioural variant (bvFTD) is characterized by the deterioration of frontal and prefrontal cortices which determine behavioural abnormalities and impairments of executive functions, working memory and social cognition [33,34,35].

Regarding PPA, its semantic variant (svPPA) is characterized by degeneration of the left anterior, middle and inferior temporal cortices [36,37]. Main cognitive symptoms of svPPA include loss of semantic memory in both verbal and non-verbal domain, impaired word comprehension and difficulties in recognizing names and faces of known people. Impairments in performing non-verbal tasks suggest the gradual disruption of the conceptual knowledge system rather than a purely language-related condition [38].

The non-fluent/agrammatic variant (nfvPPA) is characterized by cortical atrophy in the left inferior frontal gyrus, premotor cortex and anterior insula [39] and its main cognitive deficits are evident in agrammatic speech, in the comprehension of syntactically complex sentences, and the apraxia of speech, while single-word understanding and semantic knowledge are usually preserved [30,40].

The logopenic PPA (lvPPA) is characterized by atrophy of left posterior temporal cortex and inferior parietal lobe, resulting in anomia, dysfluency, impaired repetition of sentences, and impairment phonological and syntactic level of lexical processing [30] (Gorno-Tempini et al., 2011).

Parkinson’s disease (PD) is characterized mainly by motor impairment including tremor, akinesia, rigidity and postural instability. It is now well established that cognitive decline as well as difficult emotional processing is a major disabling PD symptom [41,42,43]. In many cases, impairment in the cognitive domain could be typically classified as full-blown MCI [44]. Neuropsychological assessment of cognitive domain in PD patients usually highlights mild to moderate deficits in the visuospatial domain, attention and working memory (WM) and a general decrease in executive functions [45] lead to significant behavioural symptoms too. Because these changes have a significant impact on the health care costs and quality of life (QoL) of both patients and their caregivers [46], it is a priority to identify effective intervention strategies to slow-down cognitive deterioration. To this purpose, pharmacological treatments have failed at addressing and ameliorating cognitive symptoms in patients with PD [47,48], while a series of non-pharmacological approaches, consisting in cognitive stimulation and/or non-invasive brain stimulation [49], had attracted increasing interest over the last few years.

Similar to PD, patients affected by Multiple Sclerosis (MS) are often affected by cognitive dysfunction [50,51,52], in addition to prominent motor and neuropsychiatric deficits [53]. In particular, cognitive impairment is observed in processing speed and attention, executive functions, memory and even some aspects of language [54]. Such changes have a harmful impact on the QoL of patients.

### 1.2. Neuropsychological Profiles of Main Neurodegenerative Diseases

In order to delay the onset of cognitive symptoms and slowdown cognitive decline, Computerized Cognitive Trainings and Virtual Reality revealed to be useful tools [2,55]. Computerized Cognitive Trainings (CCT) are usually based on protocols in which tasks aimed to improve and train cognitive functions are performed using electronic devices such as computers, tablets and/or smartphones [56,57,58,59].

Virtual Reality (VR) and Augmented Reality (AR) are particular kind of CCT. In VR, subjects interact with computer-generated environments which are built by researcher with the aim to control environmental variables and simulate multimodal experiences [60]. VR protocols can be differentiated by the kind of activity performed and technology adopted to provide a more or less immersive environment [61]. Non-Immersive Virtual Reality (niVR) consists of computerized protocols simulating real environment on 2D screens. In contrast, with the term fully immersive Virtual Reality (fiVR), one refers to a computer simulation that replaces the external sensorial world of the subjects with a three-dimensional artificial environment in which subjects can move or interact with as if it was a real environment [62]. This immersion can be achieved using Head Mounted Displays (HMD) or particular rooms with the artificial environment projected on the walls and motion capture devices as in the Cave Automatic Virtual Environment (CAVE) system [63]. Currently, interactions with virtual environments in VR are mediated by the adoption of devices which collect motor responses (i.e., buttons) or sensors which collect motion of body parts (i.e., motion-captures camera, accelerometers and eye-tracking), but future implementation of brain–computer interfaces as controlling devices could potentially lead to higher levels of immersion in VR [64].

In AR, additional computerized objects are simulated overlaid to the real-world environment viewed through HMD, special goggles or other devices like smartphones in order to stimulate interaction with simulated objects or integrate simulated features on real objects without the need to build a completely simulated virtual environment [65,66].

## 2. Materials and Methods

**Figure 1:**
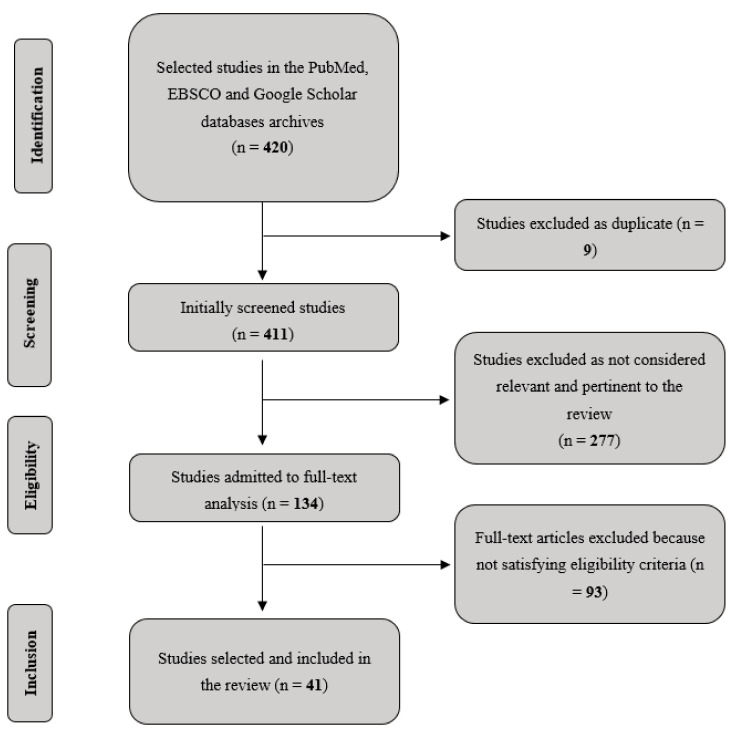
Flow-chart of the studies considered for the present scoping review.

In order to ensure reproducibility of our research, on the base of the guidelines for scoping reviews proposed by Arksey and O’Malley [67], we:

1. Identified our research question as “what is known so far from the existing literature about CCT and VR studies targeting cognitive impairment in most common neurodegenerative conditions?” In particular, we aimed to point out the scientific evidence currently available in order to provide support for health professionals to consider these promising therapeutic tools when planning rehabilitative interventions.
2. Identified relevant studies which would be as comprehensive as possible in answering our central research question. To this purpose, we adopted a strategy that involved searching for research evidence via different sources (electronic databases, reference lists, hand-searching of key journals). In a first step, we performed an EBSCO, Google Scholar and PubMed-based search using these specific combinations of keywords: “Cognitive Training” OR “Virtual Reality Training” OR “Augmented Reality Training” OR “telerehabilitation” AND ‘‘Alzheimer’s disease’’ OR ‘‘fronto-temporal dementia’’ OR ‘‘Parkinson’s disease’’ OR ‘‘Multiple Sclerosis’’. Since we were interested in exploring the latest evidence, we focused our literature search on articles that have been published between 2015 and 2020. However, we also included previously published articles whenever was necessary for clarifying the information which emerged from more recent studies. Successively, the review was further extended by considering all relevant articles reported in the references of each paper
3. Selected the studies adopting inclusion and exclusion criteria, based on our specific research question. Analysis has been primarily focused on studies clearly reporting details about cognitive training, patients’ characteristics, presence/absence of cognitive symptoms, study design and experimental protocols, quantification of training parameters of interest (in terms of length and frequency of training sessions) and brain imaging data, where available. We excluded research on healthy subjects and/or conducted in non-human animals. Finally, Duplicates and/or redundant resources across databases were removed. Figure 1 reports the followed flow-chart.
4. Charted our data, summarizing the relevant aspects of our selected studies. We recorded information as follows: Authors, Year published, Size of the sample, Diagnosis of the clinical sample, Mean age of the sample, Duration of the intervention/training, Study type, Type of experimental control condition or group, Cognitive training used, Main results, Duration and presence of a follow-up (see Table 1 and Table 2).
5. Summarized and reported our narrative account of findings, organizing the literature thematically according to a first criterion (type of neurodegenerative disorder) and an ensuring second criterion (kind CCT and VR training/intervention).

## 3. Results

### 3.1. Alzheimer’s Disease (AD)

#### 3.1.1. Computerized Cognitive Training (CCT)

Cognitive training (CT) interventions are based on protocols specifically designed to reduce degeneration of cognitive functions mainly impaired in AD, such as memory, attention, and problem-solving to restore individual global functionality [68]. Cognitive training interventions in AD are valuable. Their efficacy in delaying impairments and improving global cognitive functioning has been documented in some studies [69,70,71]. Other studies failed to show a positive effect. However, cognitive training could be problematic since poor performances could be associated with reduced therapeutic engagement, increasing frustration and depression [72].

During the last decade, easier access to electronic devices and the need for therapy from large populations had determined a wide spreading of CCT as technological aids, especially in cases of in-home therapy (or Telerehabilitation) for bedridden patients [73] or in case of pandemic events which had required social isolation [74].

CCT utilization for cognitive rehabilitation and treatment of psychiatric diseases is controversial [75,76,77,78,79]. First, there are worries about conflicts of interest from companies that advise their product to overestimate their efficacy for commercial purposes [80]. Moreover, other crucial issues in demonstrating CCT’s efficacy are the large variety of methodologies and outcome measures across studies [81], and it is still unclear whether effects on cognition are prolonged over a long-time range or can boost cognition only for a short time after the end of training [82,83]. However, a recent meta-analysis of 12 randomized controlled trials studies showed that CCT could significantly improve cognitive functions, especially in the memory domain with less extent in the attention, language and executive functions domain on patients with mild AD and MCI [84]. Different CCT protocols had been observed to be more effective on MCI and early-stage AD patients compared to other neurodegenerative conditions and healthy individuals [81,85]. Cognitive interventions targeting early-stage AD are particularly effective [68,86] since, at that stage, cognitive functions are impaired but still show residual functionality. Alescio-Lautier and colleagues [87] used computerized visual recognition memory tasks and visual focused attention tasks together with a pencil-paper semantic task with mild-AD patients. Their CCT protocols difficulty was set on each patient’s individual needs to reduce distress and maximize positive feedback that affects therapeutic outcome [72]. After 15 sessions of training (from 90 to 120 min every two weeks), they observed improvements in Mini-Mental State Examination (MMSE) scores, memory recall and verbal fluency while the control group showed a decline in performances.

Cavallo and colleagues [88] adopted a CCT protocol including tasks that targeted different cognitive functions impaired in early-stage AD (i.e., memory, attention, executive function and language) and whose difficulty was tailored to each patient’s performance. A total of 80 early-stage AD patients were recruited and divided into an active and control group. The structured CCT protocol was administered to the active group for 30 min, 3 days a week, for 12 weeks. The control group performed unstructured general computerized tasks (e.g., reading articles, listening to music, watching videos) for the same time and with the same frequency of the active group. After the training period, patients in the active group showed significant improvements in different neuropsychological measures involving different cognitive functions than the control group. This pattern was stable at the 6-months follow up. Crucially, in some tasks related to executive functions and memory, patients’ performance in the active group increased after training. In contrast, the performance of patients in the control group decreased over time. This pattern suggests that structured computerized cognitive intervention could delay cognitive decline and improve cognitive functions in early-AD patients.

Another open issue regards the duration of beneficial effects induced by CT. Most of the interventions did not last beyond six months, so that little is known about the effect of these programs in the AD patients after six months of treatment. In a recent study by Rodríguez-Mora and colleagues [89], the authors evaluate, in a sample of thirty-nine AD patients, the efficacy of the twelve-month Comprehensive Program of Cognitive Training (CPCT) consisting in a set of cognitive stimulations, intervention in activities of daily life (ADL), and motor training lasting 12 months. All patients were evaluated at baseline and in three-month intervals via the MMSE, the Cambridge Cognitive Examination (CAMCOG), the Lawton Instrumental Activities of Daily Living Scale (IADL), and the Global Deterioration Scale (GDS). The authors found no signs of mental decline between baseline and 12 months’ stage since there were no significant differences in the MMSE, IADL and GDS evaluations. On this ground, the authors concluded that the CPCT extends the benefit of non-pharmacological interventions for AD patients to twelve months and that its implementation might provide the patients’ relatives with some guarantee concerning the delay of the disease.

A crucial issue is to provide effective continuative therapies to stimulate patients’ cognition. With caregivers’ cooperation, it is possible to administrate useful, cheap, user-friendly CCT directly at home. The efficacy of daily CCT on the ability to use a tablet has been observed by Imbeault and colleagues [90]. They tailored intervention on an AD patient with severe episodic memory deficit in a single case study. The patient was trained to use the tablet to schedule fictional appointments and, finally, being tested by scheduling and participating in real ones. Moreover, the patient improved her ability to use other different applications on the tablet and reported reduced daily-life problems and improvement in memory abilities since the tablet was introduced. This study suggested the impact of portable tablet and smartphone applications on global functional improvements [91,92].

Neuropsychological improvements using another in-home CCT battery has been found by Lizio and colleagues [93]. Their protocol consisted of 7 different tasks involving different cognitive functions, including visuomotor functions, visuospatial and planning frontal executive functions, short-term visuospatial episodic memory, visuospatial attention and a central executive functioning task administered in the form of serious games [94,95]. After 15 days of daily training, participants showed improvement in accuracy and reaction times in all tasks.

Administration of CCT in the form of a memory-based iPad game has been associated with greater engagement from aMCI patients, showing robust episodic memory improvements, together with greater self-confidence, self-rating of memory ability and motivation [96]. A recent systematic review on the efficacy of in-home telerehabilitation [97] showed that its effects on cognitive abilities are comparable to conventional face-to-face interventions, highlighting the need of further research with comparable methodologies and measurements to demonstrate the in-home treatment valid alternative to classical treatments.

CT and CCT can be used alone as in telerehabilitation protocols or in combination with other therapeutic interventions to increase their effectiveness. However, the benefits associated with this combined approach depends on the treatments involved. For example, Barban and colleagues [98] used CCT for memory and executive functions combined with reminiscence therapy (RT) and found positive effects on verbal episodic memories in mild-AD, MCI and Healthy subjects and a positive effect on MMSE scores for mild-AD and MCI patients. However, improvements in functional abilities in mild-AD patients were not prolonged in the follow-up 3 months after the training and combinations with RT showed promising but not robust shreds of evidence. However, interesting positive results about a positive contribution of a computerized reminiscence therapy with the aim of a virtual partner have been documented by Lancioni and colleagues [99]. Other studies suggest a promising therapeutic approach by combining CCT and CT with neurostimulation, i.e., tDCS [100,101]; pharmacological intervention, i.e., Cholinesterase inhibitors [102]; and physical therapy [103]. Interestingly, CCT protocols administered to patients and designed to enhance specific targeted cognitive functions tailored on subjective needs are associated to greater cognitive improvements when compared to groups performing non-specific CCT protocols, placebo-control groups or classic computer games [84,104,105].

CCT related improvements could reflect cerebral neuroplasticity processes since cognitive training could improve the functionality of neural networks underlying the trained cognitive function [85,106,107,108]. In support of this hypothesis, Takeuchi and colleagues [109] found a modulation on different indices of cerebral plasticity and global cognitive functioning following 4 weeks of computerized working memory trainings in young, healthy individuals. Their findings show a large modulation of cerebral plasticity, especially over dorsolateral prefrontal cortex (DLPFC), anterior cingulate cortex (ACC) and fronto-polar regions. All these regions are neural correlates of executive functions [110], reasoning [111], emotional regulation [112] and causal reward [113], suggesting that specific CCT protocols could be particularly valuable in promoting improved functioning of regions involved in emotional and behavioural control and problem-solving, which are all functions impaired in AD patients [114,115]. Table 1 reports main information of the studies reviewed.

#### 3.1.2. Virtual Reality (VR) and Augmented Reality (AR) Trainings

In the last decades, the use of VR as a neurorehabilitation device to stimulate neuroplasticity through multisensory real-time stimulation has gained increasing attention [65]. Activities in VR can be tasks intended as simple actions specifically designed for the training of a specific cognitive function, activities which involve higher-level coordination of cognitive function and processes to complete a specific aim (i.e., cooking) or games in which subjects are engaged to reach targets by following specific rules [116]. These kinds of intervention can be implemented in both non-immersive and immersive VR. In addition, recent investigations have shown the potential of multisensory integration in fully immersive VR (fiVR) which would facilitate the formation of spatial presence, generally understood as the sense of “being there”. Such a result is achieved by adding to the user experience auditory elements such as audio-realistic sequences representing so-called “soundscapes” [117,118] and even scents which has been proven to modulate the request of cognitive resources during the processing of informative messages [119].

VR, especially with accessible, user-friendly HMD [120], video game consoles (i.e., Nintendo Wii [121]); or novel video devices such as 3d smart TV, can be helpful also for in-home telerehabilitation purposes [61]. For example, the employment of virtual reality for telerehabilitation through 3D smart TV and movement sensors allows the therapist to interact remotely with the patient and extract kinematics of motor activity for on-line feedback and off-line analysis [122]. Such electronic devices are becoming part of our daily lives, and their uses as telerehabilitation devices with stroke patients showed therapeutic outcomes similar to in-clinic intervention but lower costs both in terms of money for transportation and time spent by physical therapists [123,124].

As previously mentioned, for VR-based treatments, psychiatric symptoms such as apathy and depression are factors that could determine a poor outcome treatment [125]. For example, Manera and colleagues [126] performed a sustained attentional task in both paper-and-pencil and fully immersive VR versions. Besides the task performances, they also collected data about acceptability and satisfaction of the treatment. Their results showed that the patient with MCI and AD preferred the VR modality, and interestingly, the patient with apathetic symptoms enjoyed the experience as much as the non-apathetic patients, suggesting that fiVR could make the treatment more enjoyable and engaging, thus potentially increasing patients’ compliance and adherence to treatments [72].

Spatial navigation deficits are among the main cognitive symptoms characterizing AD from the earliest stages to the more advanced [127]. Since VR technologies can provide a strong embodiment when exploring virtual environment, this technology had been employed widely to evaluate and train spatial navigation in neurological and neuropsychiatric patients [128,129]. For example, White and Moussavi [130] performed a single-case study with an early-stage AD patient using a spatial-navigation task in fiVR three days per week for 7 weeks of training. Since older adults are not very familiar with joysticks or keyboards, they adopted a novel paradigm using a custom wheelchair as an input device to control the avatar in VR to avoid biases associated with the unfamiliarity with the classical input device (i.e., joystick and keyboard; [131]. In their spatial-navigation protocol, the participant had to navigate an artificial building to find a target previously showed on a map. After the 7 weeks of training, the participant showed improvements in navigational abilities both inside the VR environment and outside during daily living activities like driving. Neuropsychological assessment using the MoCA showed certain stability along the training period, with a slight reduction in the memory performance. Moreover, the participant showed improvements in mood and reduced self-deprecating speech, supporting the beneficial role of VR application on mood and self-efficacy [132].

Spatial computation involves navigation and the use of allocentric and egocentric coordinates to map the surrounding environment [133]. The cognitive function which allows the shifting from allocentric to egocentric coordinates is named Mental Frame Syncing, and it appears to be impaired during the early stages of AD already [134,135]. In order to promote an improvement in syncing these two coordinate systems in AD patients, Serino and colleagues used a fully immersive VR-based novel training protocol [136]. First, patients had to find target objects in a small virtual city. Then in a second stage, they had to find the previously found objects’ location starting from a different point on the map. Results showed a significant improvement in a long-term spatial memory task performance comparing the VR group with the Control group and a significant improvement in a verbal fluency performance comparing the AD patients in the VR group with a healthy controls VR group, suggesting that the VR training actually could have stimulated the mental frame syncing thus improving spatial performances.

Spatial processing is currently the most studied cognitive function with the VR technology in AD treatment. However, novel therapeutic approaches and protocols have been proposed to explore the use of such technology in order to train other cognitive functions such as attention and memory synergistically with physical activity [137] or with EEG and machine learning techniques in order to read patient’s mental state in real-time and modulate the VR task accordingly [138].

One crucial aim of VR training is to restore or preserve the ability to perform daily living activities to support patients’ autonomy and self-reliance [139,140,141]. For instance, some studies explored non-immersive VR technologies’ employment in helping AD patients re-learn cooking and demonstrated its effectiveness in reducing errors, thus limiting consequences like injuries and depression [142,143,144]. A promising novel fully immersive VR system has been recently designed by Caggianese and colleagues [145]. Their system is designed to provide a highly realistic and interactive environment in which patients can practice with instrumental activities of daily living safely and without the use of joysticks and/or keyboards. A motion tracker is used to record patients’ movements and use them as inputs making the interaction with virtual objects more similar to interaction with real ones. Interestingly, the system is composed of a fully immersive wireless HMD device for the patient and a control station for the therapist that can directly support the simulation if necessary and monitor in real-time performance and vital signals to have a clear picture of the patient’s performance and state.

An interesting application of such technology has been adopted by Quintana and Favela [146]. They used an Ambient aNnotation System (ANS) implemented in the form of a smartphone application to help patients perform efficiently daily living activities by tagging objects they are interacting with, by using textual or auditory tags in real-time. Tags can be created and edited by caregivers, and their presence is notified to the patient by an audio cue or vibration on their smartphone. This system aims to support retrieval of names, semantic and procedural information about interactive objects in the patient’s environment, reducing the impact of semantic and prospective memory deficits. More recently, a similar approach with AR for object and people recognition, geographical localization and other different features (e.g., reminders for taking medicine) for people with AD has been proposed by Kanno and colleagues [147].

Rohrback and colleagues [148] used a mixed reality commercially available device, the Microsoft HoloLens for AR, to assess AR’s supportive role in an activity of daily living involving sequential actions such as preparing tea. A total of 10 AD patients participated in the study, and their task was to prepare a cup of tea with and without the aid of AR. Their results showed that the time of execution was longer in the AR condition than the control condition without the AR device. Moreover, they only observed a trend (*p* = 0.07) for diminished sequencing errors comparing the two conditions. These mixed results show that AR could be a valuable help for patients in functionally interacting with the environment. Still, few practical aspects could negatively influence performances, such as the type of cues used in the task, the discomfort related to the wearable device’s dimensions or the additional effort induced by the interaction with a non-optimal AR interface [148].

Recently, Microsoft HoloLens has been used as a base to develop a VR system named MemHolo specifically designed for patients with AD [149]. This system is designed to train short-term and visuospatial memory to delay AD progression and obtain preliminary positive results, especially about the level of details of the simulated objects and patients’ engagement. The MemHolo system uses AR to overlay virtual object over the real environment. Users can explore the objects by simply moving their head and interact with them by voice, finger movements or a wireless clicker device depending on the task requirements. Table 2 reports main information of the studies reviewed.

### 3.2. Fronto-Temporal Dementia (FTD)

#### 3.2.1. Computerized Cognitive Training (CCT)

Currently, there is no clear evidence about the effects of non-pharmacological treatments on bvFTD. The small number of patients recruited, the lack of precise instruments for diagnosis and the overlap with early-AD behavioural symptomatology could together explain why studies about the non-pharmacological treatment of bvFTD are rare and mostly inconclusive [150]. For the same reasons, known symptom-directed treatments of bvFTD are often very similar to those used in AD, especially pharmacological ones [151,152]. Considering the quality and amount of studies existing in literature, we will focus on PPA and linguistic therapeutic approaches in the current context.

Since anomia is one of the most prominent initial symptoms of PPA, lexical-retrieval-based therapies are the treatments of choice. These approaches involve the recall of semantic or phonological features of the target items to facilitate their retrieval. Patients with svPPA are more commonly recruited as participants compared to nfvPPA and lvPPA patients, so in most of the literature about lexical retrieval, semantic-related therapies are the most employed [153,154]. However, there is different evidence about improved nouns and semantic features retrieval after training [154,155]. The period of maintenance and the generalization to untrained stimuli are still under debate.

An example of the effect of a semantic approach in different PPA subtypes has been proposed by Newhart and colleagues [156]. In their study, two older women diagnosed with svPPA and lvPPA, respectively, underwent a naming task under different cueing conditions: spontaneous naming, written naming, a notebook search, reading, repetition. Comparing naming accuracy of pre- with post-treatment assessment on trained and untrained items and categories, they observed two different patterns in the two participants. lvPPA patient showed significant improvement in naming trained and untrained items in both trained and untrained categories, whereas the svPPA patient improved in trained categories only. The degradation of semantic knowledge in svPPA could explain this differential effect of training. Moreover, these results suggest that PPA patients can improve in naming, but the generalization to untrained domains depends on each subtype’s specific pathological profile.

A different approach has been proposed by Evans and colleagues [157]. They used the open-source web platform “Anki” which was used to present multimedia flashcards containing pictures and descriptions of target items. A 72-year-old woman diagnosed with svPPA participated in the study. After each flashcard presentation, the participant had to name the target, and if she failed to name it, she could read it aloud. In any case, after the naming, she had to produce three sentences using personally relevant episodes to strengthen the connection between the target item name to semantic and contextual information. The intervention lasted 24 sessions (1 h each) over 20 months and was performed in a telerehabilitation protocol. After the treatment, the patient successfully retained 139 items and retrieved and applied some basic semantic information to perceptually dissimilar stimuli, showing a form of generalization. As also observed by Jokel and colleagues [154], most of the studies in the literature use no more than 40 trained items and generalization to untrained items is usually limited to perceptually similar items. For these reasons, the preliminary results showed by Evans and colleagues [157] are particularly interesting, suggesting that larger target database and a patient-oriented approach should be taken into consideration for future studies.

Another study using flashcard depicting personally relevant items have been recently performed [158]. In this study, eight participants with different forms of PPA (2 svPPA, 2 lvPPA, 3 nfvPPA, 1 mixed) underwent three home-based treatment phases, each followed by an assessment phase. In each treatment phase, participants were asked to name the target item based on their picture and/or written form. Not all participants showed immediate treatment-related improvements, and two of them dropped out after the first phase. The participants who showed significant treatment-specific enhancement in the early assessments maintained the positive training effect after the long-term treatment phase. However, no clear evidence about generalization has been observed. In general, this study showed that repetition and/or reading in the presence of pictures could lead to immediate treatment-related gains. Still, the lack of generalization to untrained items suggests focusing future treatments to personally and communicatively relevant items based on the single patient’s idiosyncrasies and needs.

Generalization on untrained items using a Lexical Retrieval Cascade Training for recalling semantic/episodic, orthographic and phonological information has been observed in patients with svPPA and lvPPA [159]. In this study, two different frequency of treatment sessions had been employed: one in which 10 patients performed the treatment once a week for an average of 4.7 weeks, while in the other one 8 patients performed the treatment twice a week for an average of 5.8 weeks. All participants showed increased lexical retrieval on trained items immediately and at least nine months after training regardless of the PPA sub-type. The generalization to untrained items has been associated with the systematic retrieval hierarchical strategy using residual linguistic knowledge and episodic/autobiographical information even if the involvement of episodic memory in generalization to untrained items is controversial [160].

Similar results on generalization have been observed in a single-case study involving a lvPPA patient performing a generative naming task for a total of 12 sessions in 2 weeks [161]. The training was based on the naming of different stimuli presented using visual, written and semantic cues for other categories, such as vegetables, animals, musical instruments and similar. Both trained and untrained categories have been used in the assessments. Participants showed improved ability to retrieve exemplars from both the trained and untrained categories immediately after the end of the training and at the 3 weeks’ follow-up. Interestingly, a post-treatment increased activity of the left-DLPFC observed through fMRI during a picture-naming task suggested an increased involvement of strategic planning and monitoring of lexical retrieval during the task.

Another single case study with a svPPA patient focused on verbs’ lexical retrieval after observing short videos depicting actions [162]. Baseline assessment consisted of a verb naming task and two verb comprehension tasks (a matching task and a semantic association task) on a total of 111 videos. Then, the patient received twelve training sessions in seven consecutive weeks. A total of 111 videos (mostly different from the videos used in the baseline assessment) have been divided in a cued list, uncued list and control list (37 videos each) have been used. The cued and uncued lists have been used in training and assessments, while the control list has been used only in the assessments. When evaluating videos in the cued list, the patient received feedback and cue after wrong answers accordingly to the increasing cue therapy protocol to support lexical retrieval [163] while in the uncued list no feedback or cue has been provided. Assessments were collected every four training sessions and two follow-ups after two and four weeks from the end of the training. In assessments, no cues or feedback have been provided to the patients. A significant improvement in the percentage of correct responses has been observed comparing the baseline to mid-point and follow-up assessments only in the cued list. Moreover, the percentage of correct responses was significantly higher in the cued than in the uncued and control lists at all time-points. However, no generalization to untrained verbs in the control list has been observed. This study showed that the positive effect of training on verb naming could be obtained only by providing contextual semantic-phonological cues to activate the semantic network that facilitates noun retrieval [164]. Furtherly, it also showed that such improvements could be observed after at least four weeks after the training.

In another telerehabilitation-based intervention recently published [165], two groups of patients diagnosed with svPPA and lvPPA received a lexical retrieval treatment. In contrast, the third group of patients with nfvPPA received a treatment to improve speech production and fluency through a script training protocol. All patients have mild-to-moderate cognitive and linguistic deficits. Within each group, half of the patients received the treatment in a classical face-to-face intervention. In contrast, the other half received a home-based intervention using electronic devices such as PC or tablets. All groups showed significant improvements after the training that have been maintained at 3-, 6- and 12-months follow up, and crucially, no differences between face-to-face and telerehabilitation protocols have been observed.

About novel approaches, Lavoie and colleagues [166] tested the effect of a self-administered therapy using a smart tablet in patients with svPPA and lvPPA. Trained items were selected based on interests and daily activities for each participant. Participants trained using an application named iTSA developed by authors. In the baseline assessment, naming tasks, usefulness ratings for 180 pictures and conversation tasks on specific topics were recorded. The naming task and the usefulness served to select the items that have been then included in the training. Participants performed the home-based training four days a week for four weeks, and a total of 60 items were used in each training session. They had to answer yes or no to semantic questions for each item, name it, and repeat the name after hearing a recorded voice naming the item. After the training, a post-intervention assessment and three maintenance assessments at two weeks and one and two months have been performed. All participants showed improvements in trained items, and four of them maintained these benefits at the two-month follow-up. Most of them also showed a reduction in anomia during conversation tasks. These studies suggest that self-administered telerehabilitation protocols can be valuable in promoting communicative skills.

In general, telerehabilitation protocols using CCT showed different degrees of efficacy. Improvements seem to depend on different factors such as progression of the disease, type of training, and specific patient needs. However, results are in line with a recent systematic review from Cotelli and colleagues [97]. They explored the effect of different telerehabilitation protocols on patients with AD and PPA compared to conventional face-to-face intervention. Overall, they observed that patients’ positive therapeutic outcomes were comparable to conventional face-to-face rehabilitation protocols [167]. Table 1 reports main information of the studies reviewed.

#### 3.2.2. Virtual Reality Training (VRT)

Different studies in a large variety of clinical settings explored the effect of virtual reality on the reduction of psychiatric symptoms [168], the assessment of functioning in daily living [169] or the design of personalized VR intervention with dementia patients [126,170]. However, the specific application of VR in patients with FTD is very limited. Only Burdea and colleagues [171] published a single-case study using VR with a nfvPPA patient. They adopted the BrightBrainer system, a non-immersive virtual reality environment which allows interaction through game controllers collecting arm movements and index fingers flexion. The BrightBrainer system provides different therapeutic games for training other cognitive domains such as language comprehension, executive functions, focused attention, short term visual/auditory memory and working memory. The patient underwent 16 sessions in 8 weeks. Each session’s duration increased over time from 20 to 40 min, and their level of difficulty changed based on the patient’s performance. Researchers and caregivers reported improvements in language skills, self-control, and ability to focus on a task despite decreased MMSE scores. However, further studies involving the adoption of standardized outcome measures and larger sample size involvement are required to systematically explore VR treatments’ therapeutic effect with this clinical target. Table 2 reports main information of the studies reviewed.

### 3.3. Parkinson’s Disease (PD)

#### 3.3.1. Computerized Cognitive Training (CCT)

Over the last few years, an increasing number of investigations explored the effects of CCT in PD with Randomized Control Trials (RCTs). The overall effects of these CCT over cognitive functions in PD has been partially investigated in a previous meta-analysis [172] and reviews on the topic [173,174] and could be generally resumed in a major improvement in the performance of WM, processing speed, and executive functions in PD patients who received CCT as compared to control interventions. More recently, in a study by Walton and colleagues [175], it has been shown that PD patients who received CCT to reduce the severity of their “Freezing of Gait” (FoG) symptom also improved processing speed and reduced daytime sleepiness compared to control patients. However, this not obvious pattern of positive results had been observed independently from improvements in other tests for executive functions (i.e., verbal fluency, Go/No-go task etc.) only when adjusting for the effect of dopaminergic medication. As regards to the time-related effects following CCT, it had been shown that the cognitive changes produced by cognitive trainings could be maintained at a 6-month follow-up [176] or may result in a stable improvement, preventing further cognitive decline at a 12-months follow-up [177].

Neuroimaging studies showed that the aforementioned post-treatment changes elicited by CCT might depend on changes in brain activity mainly located in fronto-parietal areas and regions of the basal ganglia [178] or to the altered functional organization of key cognitive nodes in the dorsolateral prefrontal cortex and superior parietal lobule [179]. More recently, Díez-Cirarda and colleagues [180] assessed structural and functional connectivity changes in forty-four PD patients due to a three-month integrative cognitive rehabilitation program (i.e., REHACOP). As compared to the control group, in the REHACOP group, an increase was observed in the connectivity between the left inferior temporal lobe and the bilateral dorsolateral prefrontal cortex during resting-state fMRI. Besides, in the experimental group, brain activity in the left inferior frontal lobe during the learning fMRI task was higher at post-treatment than pre-treatment. Finally, the same group showed significant and positive correlations between brain connectivity and the cognitive performance measured at post-treatment. In an analogous study by the same research group, the authors found that consistent with a progression of neurodegenerative processes, despite a series of structural brain changes. The changes observed in functional connectivity could last until 18 months’ post-treatment.

Clues on altered and improved neuronal plasticity following CCT may be found at a neurobiological level too. It had been reported that CCT in PD patients seems to produce an increase in the level of brain-derived neurotrophic factor (BDNF) [181] and variations in the dopamine transporter gene (DAT1) which granted lower striatal DAT availability and, as a consequence, a higher level of dopamine in the extracellular environment which strengthened dopaminergic pathways [182]. Table 1 reports main information of the studies reviewed.

#### 3.3.2. Virtual Reality Training (VRT)

As compared to the largely explored effects of CCT on PD-related cognitive impairment, a significantly smaller number of studies investigated the possibility to obtain benefits on the motor and cognitive symptoms of PD using trainings settled up in VR environments. Early findings come from a series of studies in which VRT were used to contrast motor symptoms in PD. In particular, a first RCT was designed to evaluate the effect of a motor-imitation training on the hypometria [183]. This is a clinical motor sign in PD that was hypothesized to rely on the dysfunction of the basal ganglia, resulting in impairment in controlling the inhibitory motor circuits [184]. In this study, sixteen patients underwent a 4-weeks VR training in which imitation of a finger-tapping task with the dominant hand was performed. The authors found that the movement amplitude increased significantly after the experimental group training for both the trained and untrained hands. The motor thresholds and silent periods evaluated with transcranial magnetic stimulation (TMS) were differently modified by training in the two groups. However, the changes in input–output recruitment were similar.

In another study by de Melo and colleagues [185], the effects of gait training with VR on walking distance and physical fitness were evaluated in a sample of thirty-seven PD patients that were randomly assigned to a control group which underwent to a conventional training (*n* = 12), a group submitted to gait training on a treadmill (*n* = 13) and a VR group submitted to gait training using the Xbox^TM^ and Kinect^TM^ (One Microsoft Way, Redmond, WA, USA) apparatus (*n* = 12). The authors found that gait training with a VR program is as effective as treadmill training with regard to gains in walking distance and improvements in temporal gait variables in individuals with PD.

According to this, in the work by Melo and colleagues, VR-training for walking skills was based on simulated walking/running by lifting the knees in a stationary march, which resulted in a constant displacement of the centre of gravity, involving symmetry, alternating actions and rhythm, which are essential to gait [186]. However, it has been recently observed that the prevalent VR locomotion techniques, i.e., walking-in-place, controller/joystick and teleportation, have peculiar aspects and differences which should be taken into account so that their distinguished interaction aspects can be documented and could guide future design process of new techniques and application to a certain clinical population. In particular, the authors of [187] have shown that the walking-in-place technique offers the highest immersion but also presents high levels of psychophysical discomfort. Controller/joystick VR locomotion is perceived as easy-to-use due to the users’ familiarity with controllers, whereas teleportation is considered to be effective due to its fast navigation and more accurate distance estimation [188], although its visual ‘jumps’ do break the users’ sense of immersion (see also Cherep and colleagues for a specific investigation on the teleportation technique [189]).

More recently, a “virtual reality (VR) based” gait manipulation strategy has been proposed to improve gait symmetry by equalizing step length [190]. In this study, fifteen PD patients with FoG were assessed on a GAITRite^®^ (CIR System, Inc., Franklin, NJ, USA) walkway. Natural gait was compared with walking conditions during “VR-based” gait modulation tasks to equalise gait symmetry using visual or proprioceptive signals. Compared to natural gait, VR manipulation tasks significantly increased step width and swing time variability for both body sides. Within the VR conditions, only the task with “proprioceptive-visual dissociation” by an artificial backward shifting of the foot improved spatial asymmetry significantly with both sides’ comparable step lengths. Specific, hypothesis-driven VR tasks represent an efficient tool to manipulate gait features as gait symmetry in PD, potentially preventing FOG.

Regarding VR trainings aimed at improving cognitive aspects in PD, to the best of our knowledge, the first evidence comes from a study investigating the influence of a 6-months treatment with non-immersive VR (niVR) using the Xbox360^TM^ and Kinect^TM^ games, on the QoL of people with PD [191]. The authors reported a statistically significant difference after 3 months of treatment with the niVR games regarding mobility, emotional well-being, stigma, cognition, and total score of the Parkinson’s Disease Questionnaire (PDQ-39). After 6 months of treatment, the results were maintained, but no further improvements were observed. In a more recent study, the effects of a VR training with BTS Nirvana (BTS-N) system in the cognitive and behavioural recovery were evaluated in patients with PD [192]. BTS-N is a semi-immersive therapy system that allows creating virtual scenarios with which the patient may interact. Twenty PD patients were recruited and undergo to 8-weeks neuro-rehabilitation programme consisting of 24 sessions with BTS-N or traditional cognitive training. Patients who underwent the VR training showed a greater improvement in cognitive functioning, with particular regard to executive functions and visuospatial abilities (both evaluated through Addenbrooke Cognitive Examination–Revised—ACE-R and MMSE), as compared with the control group. Table 2 reports main information of the studies reviewed.

### 3.4. Multiple Sclerosis (MS)

#### 3.4.1. Computerized Cognitive Training (CCT)

Over the last years, many studies have assessed the efficacy of training for the amelioration of cognitive deficits in MS. Their results were summarised in two previous reviews on the topic [193,194,195]. These showed that, differently from early interventions based on learning and memory tasks, the focus of more recent approaches has moved to other domains such as executive function and attention. Duration of CT in MS could vary from one day up to 6 months with frequencies of intervention sessions ranging from twice per month to five times per week. Due to these notable differences, independent studies come to contrasting results regarding the specific effect related to the timing in treatment intensity [193]. Nonetheless, the efficacy of computer-assisted training with a brief duration of 3 months has been recently proved [196,197]. More specifically, in a randomized controlled study on 62 MS patients with mild-to-moderate levels of cognitive impairment, at post-training, Perez-Martin and colleagues showed significant improvements in verbal memory, working memory and phonemic fluency in the experimental group. Furthermore, reduction in anxiety symptoms and significant improvement in quality of life were observed. No such effects were found in the control group.

Similarly, Charvet and colleagues [197] used an adaptive, computer-based training with the same 3-month duration. This “tele-rehabilitation” programme was implemented on a laptop and accessed from home, with remote supervision. This training was proved to improve general cognitive functioning (with particular reference to WM and executive functions) in MS. Its characteristics allow for rapid recruitment and high compliance and can be readily applied to other neurological conditions associated with cognitive dysfunction [197].

In general, as regards the post-treatment effects on the cognitive domain, it has been shown that independently on the duration of CT, “non-specific” rehabilitation programmes led to weaker effects [198], as compared to focused cognitive trainings that are specifically designed to act on one cognitive function as for example attention or speed processing [199,200]. Such differences between “general” and “focused” CTs also reflect on the modulation of brain activity as shown by a series of early neuroimaging investigations which pointed out that, following cognitive rehabilitation, a series of changes in brain activation were found in fronto-temporal regions and the cerebellum [201,202]. The increase in brain activation was interpreted as a mechanism to compensate for the cognitive deficits [203,204] while the failure of such mechanisms would rather lead to cognitive deterioration [205].

In contrast, it was found that intensive CCT specifically focused on the rehabilitation of attention and information processing in MS patients produced an increase in brain activity over right posterior cerebellar lobule [206] and superior parietal and frontal cortex [206,207] during the execution of a Stroop Task. Most important, using resting-state fMRI, it was also observed that focused CCT produced a significant increase in functional connectivity of several cognitive-related resting-state networks (e.g., anterior cingulate cortex, prefrontal cortex and posterior cingulate cortex) in the treatment group though not in the control group, where in contrast, a decrease in the functional connectivity of the same networks was observed [207]. Recent results also showed that changes in resting-state functional connectivity of cognitive-related networks at a six-month follow-up could be based on the persistence of positive effects induced by cognitive rehabilitation [208]. Overall, these studies demonstrated that an intensive programme of stimulation, particularly the ones focused on attention and executive functions, could contrast cognitive decline and affect neural plasticity.

More recently, an interesting study [209] investigated the effects of training on MS patients’ neuroplasticity using a new and non-conventional neuroimaging technique defined as magnetic resonance elastography (MRE) [210]. MRE is a non-invasive imaging technique that provides information on brain tissue health by measuring their mechanical properties [211,212,213]. In the study by Sandroff and colleagues [209], the authors specifically examined the effect of a 12-weeks supervised aerobic exercise training on learning and memory and the effects on the hippocampal viscoelasticity. Results showed, at post-training small-to-moderate effects on learning and memory abilities measured with CVLT-II. Nonetheless, it was found a large intervention effect on hippocampal viscoelastic properties. Notably, measures of viscoelasticity in the hippocampus were also strongly related to scores in the memory performance.

This evidence suggests that CCT might improve MS patients’ cognitive abilities, mainly whether focused training is implemented. By the way, no definite conclusions can still be drawn about the effects of rehabilitation outcomes. Larger studies with bigger samples and longer follow-up periods are needed to generalize these results and verify whether these cognitive rehabilitation treatment effects persist over time. Table 1 reports main information of the studies reviewed.

**Table 1:** Main information of the reviewed CCT experimental studies.

Number, Authors, Published Year	Sample (*n*)	Diagnosis	Mean Age (Years) (SD)	Duration (Days × Weeks)	Study Type	Control	Cognitive Training Used	Main Results	Duration Post-Treatment
Alescio-Lautier et al. (2019) [87]	12	AD	81 1.68	15 sessions	RCT	Control group	Memory, attention and semantic tasks	Increased memory recall and verbal fluency	Not tested
Cavallo et al. (2016) [88]	80	AD	76.5 2.88	3 d × 12 w	RCT	Control group	Memory, attention, EF and language tasks	Improvement in different neuropsychological domains	6 months
Rodriguez-Mora et al. (2020) [89]	39	AD	76.31 7.17	5 d × 12 months	Pilot study	None	Different cognitive trainings, ADL and motor tasks	Arrested decline in all tested functions	Not tested
Imbeault et al. (2018) [90]	1	AD	65	2 d × 8 w + 23 sessions	Single-case	None	Prospective memory task on tablet (telerehabilitation)	Improved ADL and memory abilities	Not tested
Lizio et al. (2019) [93]	15	AD	69.7 0.8	7 d × 2 w	Pilot study	None	Spatial abilities, EF and memory tasks on tablet (telerehabilitation)	Increased accuracy and reduced reaction times in all domains	Not tested
Savulich et al. (2017) [96]	42	aMCI	75.2 7.4	8 sessions	RCT	Control group	Memory and visuospatial game on iPad	Increased episodic memory and visuospatial abilities	Not tested
Barban et al. (2016) [98]	348	AD, MCI and HE	77 5.7	2 d × 3 months	Crossover RCT	Control group	Different cognitive functions + RT	Increased MMSE scoring	None
Newhart et al. (2009) [156]	2	lvFTD, svFTD	65/60	~25 sessions	Proof-of-concept study	None	Cueing hierarchy naming treatment	Increased naming performances on treated items in both subjects and also in untreated items in lvFTD one	Not tested
Evans et al. (2016) [157]	1	svFTD	72	24 sessions (20 months)	Single-case	None	Flashcard naming task (telerehabilitation)	Increased naming performances	Not tested
Croot et al. (2019) [158]	8	Various PPA	64.8 5.9	2 w + 2 w + 26 w	Single-Case Experimental Design	None	Repetition and reading with cueing pictures (telerehabilitation)	Mixed results, 3 subjects showed increased picture naming performances	Up to 6 months
Henry et al. (2019) [159]	18	lvFTD, svFTD	65.2 8.3	1 d × 4–8 w/2 d × 4–8 w	Clinical Trial	None	LRCT	Increased naming on trained and untrained items	1 year for trained and 6 months for untrained items
Beeson et al. (2011) [161]	1	lvPPA	77	6 d × 2 w	Single-case	None	Generative naming task	Improved word retreival on trained and untrained items	6 months
Macoir et al. (2015) [162]	1	svFTD	72	5 d × 2 w	Single-case	None	Video-cued action naming task	Increased naming on trained actions	4 weeks
Dial et al. (2019) [165]	31	lvFTD, svFTD, nfvFTD	~65 ~8	(not clearly reported)	Clinical Trial	None	LRCT or VISTA (telerehabilitation or face-to-face)	Increased primary outcomes (word retrieval or fluency); no differences between telerehabilitation and face-to-face interventions	12 months
Lavoie et al. (2019) [166]	5	lvFTS, svFTD	72.2 5.4	4 d × 4 w	Single-case	None	Functional Vocabulary Treatment (telerehabilitation)	Increased naming for trained items and reduced anomia in natural conversation	2 months
Walton et al. (2018) [175]	65	PD	~68 ~8	2 d × 7 w	RCT	Active control group	Battery with different cognitive trainings	Reduced FoG and increased processing speed	Not tested
Sinforiani et al. (2004) [176]	20	PD	68.9 7.1	2 d × 6 w	Pilot study	None	Attention, abstract reasoning and visuospatial training	Increased verbal fluency, logic memory and Raven’s matrices	6 months
Petrelli et al. (2015) [177]	47	Non-demented PD	~69 ~9	2 d × 6 w	RCT	Control group	Attention, memory and EF tasks	Reduced cognitive decline	12 months
Diez-Cirarda et al. (2017) [180]	15	PD	66.07 4.8	3 d × 13 w	Clinical Trial	None	Attention, memory and EF tasks	Increased cognitive performances and increased functional connectivity	18 months
Perez-Martin et al. (2017) [196]	62	MS	44.9 9.8	12 sessions	RCT	Control group	Training of several cognitive domains (telerehabilitation)	Increased memory, verbal fluency and reduced anxiety	Not tested
Charvet et al. (2017) [197]	135	MS	50 12	5 d × 12 w	RCT	Active control group	Training of several cognitive domains (telerehabilitation)	Increased cognitive functions	Not tested
Brissart et al. (2013) [198]	20	MS	42.5 5.1	13 sessions	RCT	Control group	Training of several cognitive domains	Increased verbal, working memory and verbal fluency performances	Not tested
Mattioli et al. (2010) [199]	150	MS	41–53	3 d × 12 w	RCT	Control group	Attention, Information Processing, EF trainings	Increase in all cognitive functions and reduced depression	Not tested
Fink et al. (2010) [200]	50	MS	44.8 8.2	4–5 d × 6 w	RCT	Control group	EF or visual CCT trainings	Increased EF and verbal learning	12 months
Cerasa et al. (2013) [206]	23	MS	31.7 9.2	2 d × 6 w	RCT	Control group	Different attentional trainings	Increased attentional abilities and SPL activity	Not tested
Filippi et al. (2012) [207]	20	MS	46.7	3 d × 12 w	RCT	Control group	Attention, Information Processing, EF trainings	Increased cognitive functions and increased activity in fronto-parietal regions	Not tested
Parisi et al. (2014) [208]	18	MS	43.6	12 weeks	RCT	Control group	Attention, Information Processing, EF trainings	Increased cognitive functions and changes in FC	6 months
Sandroff et al. (2017) [209]	8	MS	43.5 10	3 d × 12 w	Pilot RCT	Control group	Treadmill walking	Increased learning and memory abilities and related changes in hippocampal viscoelastic properties	Not tested

AD = Alzheimer’s Disease; ADL = Activities on Daily Living; aMCI = amnestic Mild Cognitive Impairments; CCT = Computerized Cognitive Training; EF = Executive Functions; FC = Functional Connectivity FoG = Freezing of Gait; HE = Healthy Elderly; LRCT = Lexical Retrieval Cascade Treatment; lvFTD = logopenic variant of frontotemporal dementia; MS = Multiple Sclerosis; PD = Parkinson’s Disease; PPA = Primary Progressive Aphasia; RT = reminiscence therapy; SPL = Superior Parietal Lobule; svFTD = semantic variant of frontotemporal dementia; VISTA = Video Implemented Script Training for Aphasia.

#### 3.4.2. Virtual Reality Training (VRT)

Exercises and motor programs for rehabilitation in MS and patients with other neurological diseases usually require that qualified professionals supervise patients’ performance and be performed in specific neuro-motor rehabilitation structures. In order to overcome these difficulties, over the last years, there has been an increase in the number of investigations trying to assess the efficacy of Virtual reality (VR) in motor assessment and rehabilitation [214]. As shown by the use of VR-training in strokes, this allows the repetitive practice, feedback information and higher performance in sensory, cognitive and motor domains [215]. In addition, VR interfaces permit to create a number of different environments for rehabilitation exercises and track each patient’s performance [216].

In VR-applications for the rehabilitation of patients with MS, results from a series of studies are well resumed in a previous review by Massetti and colleagues [217] and more recently in a systematic review and meta-analysis by Casuso-Holgado and colleagues [218]. They showed that VR trainings in MS patients produced improvement in the following: (1) balance performance measured as a function of the fall risk and postural stability tests [216,219,220], and furthermore, results of VR rehabilitation and tele-rehabilitation programs resulted in optimized sensory information processing and integration systems of information necessary to maintain balance and postural control and that also allows anticipatory postural control and response mechanisms [221,222]; (2) gait [223], with particular reference to speed and stride length [224] (for a combined treadmill and VR trainings see [225]); (3) arm movement and control of motor planning during “reaching” in the treated arm [226] and the contralateral limb [227].

Taken together, studies investigating the efficacy of VR trainings in MS (alone or combined with other technology) demonstrated efficient results and could represent a valid therapeutic alternative to traditional motor rehabilitation [221,222]. However, some concerns directly connected to the use of VR, such as excessive fatigue, game difficulty level and high physical requirements should be considered and evaluated as important factors in future studies.

To summarize, VR training could be considered at least as effective as conventional training and more effective than no intervention to treat balance and gait impairments in multiple sclerosis rehabilitation; overall, the use of VR in motor and cognitive rehabilitation of MS showed promising results, but follow-up studies are needed to enhance treatment effects in patients with MS.

## 4. Conclusions

The major neurodegenerative disorders here considered are heterogeneous in their clinical profiles and underlying pathophysiology. In the vast majority of cases, patients share the presence of significant cognitive impairment, depending on the disease itself and on the clinical staging achieved at that point in time. Due to the lack of effective pharmacological treatments for most prominent cognitive symptoms, researchers and clinicians urgently need valid tools to contrast patients’ cognitive decay. The dramatic experience of the current pandemic has shown and continues to present a significant negative impact on the continuity of care of patients not affected by COVID-19, thus reducing the resources available in hospitals and clinics for standard rehabilitation treatments of cognitive symptoms in neurodegenerative diseases.

It has been demonstrated that CCT seems to be particularly effective in preventing cognitive decline in the healthy ageing population [81] and in MCI [228,229,230]. On these grounds, the application of CCT and VRT in patients with cognitive impairment had started to show interesting evidence in recent years. The two important issues at hand pertain to the possibility for patients to acquire new procedural skills (and not just keep those acquired in the distant past) and to maintain them once the training comes to an end. In most of the studies reviewed here, after the cognitive intervention, the authors were able to see a significant effect of them on different neuropsychological measures. In some of these studies, the intervention itself was able to reach a significant positive impact on functional measures too, thus really suggesting the possibility for cognitive interventions to make a difference in patients’ life via the improvement of how they tackle everyday life tasks and activities.

The present in-depth review strongly suggests that CCT and VR tools can represent an effective therapeutic option for treating cognitive deficits in neurodegenerative conditions, especially during these difficult times when the access to regular hospital consultations are not easily provided. On this ground and with a particular view to the future and technology development, we see as very promising those protocols which, with caregivers’ cooperation, allow to administrate user-friendly CCT directly at home, using portable smart devices such as tablet and smartphones (see, for example, 90, 91, 96). At this point in time, the majority of studies reviewed here showed a significant clinical effect of cognitive training (please refer to Table 1 and Table 2 that showed a significant improvement of patients in 36 out of 41 studies considered). In addition, a significant proportion of CCT studies can be considered robust in methodological terms (e.g., 15 out of 28 were RCTs). By referring to a widely accepted classifications of efficacy (e.g., Grade Practice Recommendations), CCT should be considered a recommended therapeutic option, meaning that the qualifying evidence can be classified as levels II, III or IV, and findings are generally consistent across studies. In terms of implications for clinical practice, clinicians should follow a recommendation but should remain alert to new information and sensitive to patients’ preferences.

**Table 2:** Main information of the reviewed VR experimental studies.

Number, Authors, Published Year	Sample (*n*)	Diagnosis	Mean Age (Years) (SD)	Duration (Days × Weeks)	Study Type	Control	Virtual-Reality Training Used	Main Results	Duration Post-Treatment
Manera et al. (2016) [126]	57	MCI/AD	75.6 7	1 session	Feasibility study	VR task vs. Paper-pencil task	Attentional task (Selective and sustained attention)	Increased satisfaction and preference to VR-task	Not tested
White and Moussavi (2016) [130]	1	AD	74	1 d × 7 w	Case study	None	Virtual Reality Navigation environment	Improved navigation skill	5 weeks/28 weeks
Serino et al. (2017) [136]	20	AD	87.6 4.8	3 d × 3/4 w	Development-of-Concept Trial	Control Group	VR-training for spatial abilities	Improved long-term Spatial memory	Not tested
Caggianese et al. (2018) [145]	-	-	-	-	Project study	-	VR for realistic enviroment	-	Not tested
Quintana and Favela (2012) [146]	6	Healthy subjects	28	1 session	Project study	None	Ambient aNnotation System (ANS)	Improved recognition of tags with audio notifications	Not tested
Rohrbach et al. (2019) [148]	10	AD	71.8 11.1	1 session	Crossover study	AR condition vs. Natural condition	Therapy Lens (Microsoft Hololens^TM^)	Trend in diminished sequencing errors	Not tested
Aruanno and Garzotto (2019) [149]	11	MCI	84.1 7.2	1 session	Feasibility study	None	MemHolo (Mixed Reality HoloLens^TM^)	Positive evaluation of MemHolo	Not tested
Burdea et al. (2015) [164]	1	PPA	51	2 d × 8 w	Single-case study	None	BrightBrainer^TM^	Improved verbal skills	Not tested
Robles-Garcia et al. (2016) [183]	16	PD	66.6 9.5	4 w	Randomized controlled pilot-study	Active-control group	VR-Motor imitation	Decreased hypometria	Not tested
de Melo et al. (2018) [185]	37	PD	62.2 10.6	3 d × 4 w	Randomized, controlled clinical study	Control group, Treadmill group	VR-Gait training	Improved gait	30 days
Janeh et al. (2019) [190]	15	PD	67.6 7	1 session	Pilot study	Natural gait vs. VR-gait	VR-Gait training (GAITRite^TM^; CIR Systems, Inc., Franklin, NJ, USA)	Improved gait	Not tested
de Menezes Sanguinet et al. (2016) [191]	14	PD	64 9	6 m	Uncontrolled clinical study	None	Non-immersive virtual reality games with Kinect^TM^ (One Microsoft Way, Redmond, WA, USA)	Improved PDQ-39 scores and mobility/cognitive skills	Not tested
Maggio et al. (2018) [192]	20	PD	69.4 8.2	3 d × 8 w	Randomized Clinical study	Control group	BTS Nirvana (BTS-N)	Improved cognitive function	Not tested

AD = Alzheimer’s disease; AR = Augmented reality; MCI = Mild Cognitive Impairment; PD = Parkinson’s disease; PPA = primary progressive aphasia; VR = Virtual Reality.

Regarding VR studies, the difficulty in recruiting a large sample of patients and planning RCTs makes its results less strong, which should be considered as a promising but preliminary evidence. At this point in time, the level of recommendation considers VR tools a viable therapeutic option, meaning that the qualifying evidence can be classified as levels II, III or IV with findings not always consistent across all studies. However, in our view, larger and more robust studies would help to overcome some of the limitations that small-scale VR studies currently present. In doing so, a more evidence-based clinical reasoning will permit to consider seriously the use of these tools when targeting cognitive deficits in neurodegenerative disorders. Despite these limitations, the role of VR and AR tools in ameliorating QoL of neurodegenerative patients should not be cancelled out. In particular, we refer to some AR application treated in this review [146,147,148,149] which even if could not be considered as a form of therapeutic intervention, still provides the potential to become in the future a useful tool for the everyday life of patients and caregivers, allowing to supplement with technology the impaired skills of these patients.

To conclude, a lot of work still needs to be done. Further investigations need firstly to establish reliable stimulation parameters (e.g., number of cognitive sessions per week, duration of each session and length of treatments) that may induce potentially more and long-lasting beneficial outcomes. This point becomes particularly relevant in order to create standardized and shared protocols of clinical interventions, differentiated according to the type or the stage (prodromal, initial or advanced) of the different neurodegenerative diseases. Secondly, future studies should foster our knowledge of how patients’ achievements could be maintained for as long as possible after the end of the training. To this aim, all of the works reviewed here, which included in their experimental design a follow-up evaluation, are to be looked at as a reference point. Last but not least, further investigations should identify the amount of learning that patients affected by neurodegenerative conditions can fruitfully achieve and the degree of generalizability of their newly acquired skills to everyday life tasks.
